# Supplementary material for: Clinicopathologic Characteristics Associated with Prognosis in Ocular Extranodal Marginal Zone B Cell Lymphoma
Source: Medicina (Kaunas). 2022 Jun 17;58(6):818. doi: 10.3390/medicina58060818 (PMC9229471; doi:10.3390/medicina58060818)
Supplement: Supplementary file 1 [file medicina-58-00818-s001.zip › medicina-1719651-supplementary.pdf]

**Supplementary Materials:**

**Table S1.** TNM-based AJCC staging system for ocular adnexal lymphoma, eighth edition

| Category | TNM Criteria |
|---|---|
| Primary tumor (T) | |
| T0 | No evidence of lymphoma |
| T1 | Lymphoma involving the conjunctiva alone without eyelid or orbital involvement |
| T2 | Lymphoma with orbital involvement with or without conjunctival involvement |
| T3 | Lymphoma with preseptal eyelid involvement with or without orbital involvement |
| T4 | Orbital adnexal lymphoma and extraorbital lymphoma extending beyond the orbit to adjacent structure, such as bone, maxillofacial sinuses, and brain |
| Lymph node involvement (N) | |
| N0 | No evidence of lymph node involvement |
| N1 | Involvement of lymph node region or regions draining the ocular adnexal structures and superior to the mediastinum (preauricular, parotid, submandibular, and cervical nodes) |
| N1a | Involvement of a single lymph node region superior to the mediastinum |
| N1b | Involvement of two or more lymph node regions, superior to the mediastinum |
| N2 | Involvement of lymph node regions of mediastinum |
| N3 | Diffuse or disseminated involvement of peripheral and central lymph node regions |
| Distant metastasis (M) | |
| M0 | No evidence of involvement of other extranodal sites |
| M1a | Noncontiguous involvement of tissues or organ external to the ocular adnexa (e.g., parotid glands, submandibular gland, lung, liver, spleen, kidney, breast) |
| M1b | Lymphomatous involvement of the bone marrow |
| M1c | Both M1a and M1b involvement |

**Table S2.** Antibodies used for immunohistochemistry

| Antibody | Company | Dilution rate |
|---|---|---|
| CD20 | DAKO, Santa Clara, CA, US | 1:500 |
| CD3 | DAKO, Santa Clara, CA, US | 1:250 |
| Bcl-2 | DAKO, Santa Clara, CA, US | 1:300 |
| CD10 | LEICA, Buffalo Grove, IL, US | 1:50 |
| Cyclin D1 | LEICA, Buffalo Grove, IL, US | 1:50 |
| Ki-67 | DAKO, Santa Clara, CA, US | 1:300 |
| IgG | Cell Marque, Darmstadt, Germany | 1:300 |
| IgG4 | Cell Marque, Darmstadt, Germany | 1:1200 |

**Table S3.** Univariable analysis of prognostic factors

| Variable | Response, n (%) | | p-value |
|---|---|---|---|
| | CR (n=33) | Non-CR (n=15) | |
| LDH >225 U/L | 13 (39.4%) | 10 (76.9%) | 0.022* |
| Ki-67 ≥ 5% | 25 (75.8%) | 9 (60.0%) | 0.266 |
| Treatment (RT or CT with immunotherapy) | 26 (78.8%) | 7 (46.7%) | 0.026* |
| $SUV_{max}$ | 2.52 (1.30) | 2.64 (1.78) | 0.822 |

*$p < 0.05$, Chi-square test and Mann–-Whitney test are used. Values are presented with n (%) or mean (standard variation).
